# Response to comments on “Evolutionary transitions between beneficial and phytopathogenic *Rhodococcus* challenge disease management”

**DOI:** 10.7554/eLife.35852

**Published:** 2018-05-08

**Authors:** Jeff H Chang, Melodie L Putnam, Niklaus J Grünwald, Elizabeth A Savory, Skylar L Fuller, Alexandra J Weisberg

**Affiliations:** 1 Department of Botany and Plant PathologyOregon State UniversityCorvallisUnited States; 2 Center for Genome Research and BiocomputingOregon State UniversityCorvallisUnited States; 3 Molecular and Cellular Biology ProgramOregon State UniversityCorvallisUnited States; 4 Department of AgricultureAgricultural Research ServiceCorvallisUnited States; University of ChicagoUnited States

**Keywords:** *Rhodoococcus*, pistachio bushy top syndrome, diagnosis, *R. fascians*, None

## Abstract

Randall et al., 2018 and Vereecke, 2018 have raised concerns about a paper we published (Savory et al., 2017). Here, we respond to those concerns.

## Potential misdiagnosis of an epidemic; Presence of virulence genes (Randall et al., 2018)

We questioned whether Stamler et al., 2015a adequately fulfilled Koch’s postulates, which is central to establishing the etiology of disease.

First, high doses of PBTS1 and PBTS2 could affect the growth of UCB-1 pistachio (Stamler et al., 2015a). However, the effects observed upon inoculation were not the same as the diverse symptoms observed in fields.

Second, Stamler et al., 2015a relied on the color and morphology of colonies to identify pathogenic *Rhodococcus* and infer their abundance in orchards.

Third, amplification of *vicA* was used to demonstrate the bacteria re-isolated from greenhouse-infected plants were identical to PBTS1 and PBTS2 (Stamler et al., 2015a). It is unclear how PBTS1 could have been verified. When using primers with the same sequences as those used by Stamler et al., 2015a, we could not amplify *vicA* of PBTS1 (Savory et al., 2017). The *vicA* allele deposited by Stamler et al., 2015a does not align to the genome sequence of PBTS1, from which it was reportedly amplified (KP274063.1; Savory et al., 2017). Results in a Masters thesis by a student at Ghent University supervised by Drs. Randall and Vereecke showed that amplification of *vicA* from PBTS1 is not reproducible (Deckers, 2017).

Fourth, *vicA* is recommended and used to inform on the presence of pathogenic *Rhodococcus* (Randall, 2015a, 2015b; Stamler et al., 2015a, 2015b). This gene does not discriminate between pathogenic and non-pathogenic genotypes (Savory et al., 2017).

Fifth, Randall et al., 2018 stated that *Rhodococcus* isolated from UCB-1 pistachio ‘contain a subpopulation of bacterial cells with virulence genes.’ Testing a mixed culture goes against the intent of Koch’s postulates, which recommend the isolated microorganism to be grown in pure culture.

Sixth, our tests using pure cultures of PBTS1 and PBTS2 failed to reproduce previous findings (Stamler et al., 2015a; Savory et al., 2017). We did not observe disease symptoms on UCB-1 pistachio, *Nicotiana benthamiana*, or pea; the latter two are commonly used susceptible hosts for testing phytopathogenic *Rhodococcus*. We also tested other isolates that lack virulence genes, and none could cause disease symptoms. In a key experiment, we demonstrated that when isolates, including PBTS2, were modified to carry a virulence plasmid, they caused disease to *N. benthamiana*.

Seventh, to cause disease, phytopathogenic *Rhodococcus* require the *fas* genes, which are frequently carried on a plasmid. Stamler et al., 2015a described the successful amplification of *fas* genes from PBTS1 and PBTS2. However, the deposited sequences are from two small regions of *fasD* and are joined with 280 Ns (KP274064.1, KP274067.1). A different fragment of *fasD* amplified from four additional pistachio-associated isolates was described separately (Stamler et al., 2015b). The only available sequence of one of the amplified products aligns distal to the regions where the primers anneal (KR153288; Stamler et al., 2015b).

Both ourselves and the Masters student at Ghent were unable to amplify *fasD* from DNA derived from pure cultures of wild type PBTS1 or PBTS2 (Savory et al., 2017; Deckers, 2017). The thesis reported on the use of primers similar in sequence to those used by Stamler et al., 2015a and designed for amplifying *fasD*. The thesis further reported that use of the primers led to the false amplification of products from DNA purified from isolates demonstrably lacking virulence plasmids and PBTS1 (aka JK1) and PBTS2 (aka SR13). The thesis concludes: ‘Therefore, for these four primers [primer pairs for *vicA*, pFi_09, *attA*, and *fasD*], the results obtained for the isolates are unreliable.’

Last, Stamler et al., 2015a, 2015b did not report data on the amplification of genes or report data that initially led them to *Rhodococcus*. Stamler et al., 2015a described direct sequencing of 16S rDNA amplified from total DNA isolated from UCB-1 rootstocks showing the syndrome. The sequences of the products were described as having 89% to 99% identity to 16S rDNA of *Rhodococcus*. It is unclear how products corresponding to single genotypes of *Rhodococcus* could be amplified in reactions consisting of universal primers that target conserved regions of 16S rDNA and templates with microbial community DNA (Yang et al., 2002). In addition, the universal primers match perfectly to diverse chloroplast genome sequences and their use will preferentially amplify the plastid genome (Randall, 2015a, slide 66).

Randall et al., 2018 states: ‘the statements regarding tree removal…are inaccurate’. This is inconsistent with an article published in the *Western Farm Press* on February 19, 2015 that quotes Dr. Randall (Northcutt, 2015). The following extract is taken from this article: 'some growers are responding to PBTS [pistachio bushy top syndrome] in their fields by removing entire orchards and bearing the extensive costs of replanting with new trees. Others are removing only those trees showing signs of PBTS and replanting with new trees. However, Randall cautions... "We’ve detected *Rhodococcus* on trees that looked healthy in some infected orchards and, when we’ve gone back later to check, we’ve found these trees had developed PBTS symptoms", she says’.

The preponderance of evidence published in Savory et al. (2017) and described herein, are consistent with the likelihood that non-pathogenic *Rhodococcus* isolates were misdiagnosed as the causative agent of pistachio bushy top syndrome.

## Subpopulations and loss of detectable virulence genes in PBTS *Rhodococcus* isolates when culturing on synthetic media (Randall et al., 2018); Appropriateness of diagnostic tools (Vereecke, 2018)

If the plasmid harboring the virulence gene is unstable, it is unclear how *fasD* fragments could have been amplified from the pistachio-associated isolates in the original studies (Stamler et al., 2015a, 2015b). Similarly, it is unclear how pure cultures of PBTS1 and PBTS2 were grown to fulfill Koch’s postulates. Detailed descriptions of the methods that were used to grow *Rhodococcus* and the data that confirmed the plasmids were maintained during culturing should be provided.

Randall et al., 2018 states that the published genome sequences of PBTS1 and PBTS2 ‘concerned draft sequences’. This argument fails to explain why *fasA, fasD*, or the virulence plasmid-specific pFi_09 gene cannot be amplified from the isolates. It is also confusing because both genomes were sequenced using a PacBio, the deposited sequences have assembly levels of ‘Complete Genome’, and each has a single assembled chromosome contig (CP015219 and CP015220; Stamler et al., 2016).

Conclusions stated in Nikolaeva et al., 2009 were used to support the plasmid instability hypothesis (Randall et al., 2018; Vereecke, 2018). Nikolaeva et al. demonstrated that plants are hosts to mixed populations of *Rhodococcus* but did not show that colonies were genetically homogenous, nor did they directly test whether plasmids were lost from *Rhodococcus* grown in pure culture or after growth *in planta*. We cultured *Rhodococcus* isolates and successfully sequenced 64 plasmids (Creason et al., 2014; Savory et al., 2017). The results of Nikolaeva et al., 2009 are consistent with ours showing bacteria isolated from plants are a mixed population that can be polymorphic for the plasmid (Savory et al., 2017).

## High plasmid instability in *R. fascians* (Randall et al., 2018)

Randall et al., 2018 suggest that in Savory et al. (2017), we stated that Nikolaeva et al., 2009 used the ‘wrong molecular tools to evaluate pathogenicity’. This is a misunderstanding. We suggested that *vicA*, as used in Stamler et al., 2015a, 2015b is not appropriate for diagnosing pathogenic *Rhodococcus*.

## Poor growth of the control pistachio UCB-1 trees (Randall et al., 2018)

It is not valid to compare between the datasets to infer on the health of plants. Treated plants were compared to untreated plants grown in the same environment. Our assays were done in greenhouses in Oregon (44° N, 123° W) whereas in previously published work (Stamler et al., 2015a), plants were assayed in a different year and in New Mexico (32° N, 106° W). We fertilized the plants when necessary and periodically inspected the plants and saw no signs that their health was compromised. It is also important to note that our experiments included negative and positive controls, findings were consistent across all genotypes of *Rhodococcus* and species of plants tested, and results were repeatable.

## Plant growth promotion by the *Rhodococcus* pistachio bushy top syndrome (PBTS) isolates (Randall et al., 2018)

We hypothesized the changes are beneficial because root hairs increase surface area. Furthermore, in a talk that is available online, Elizabeth Fichtner has reported that inoculation of *Rhodococcus* originally cultured from pistachio had significant plant growth promoting effects on pomegranate (Fichtner, 2017; comments at ~14 min 11 s)

## Misrepresentation of previous work (Randall et al., 2018)

Points i and iii are addressed above.

In point ii, Randall et al., 2018 suggest that published observations showing pathogens can be isolated from asymptomatic tissue support the findings by Stamler et al., 2015a, which relied on culturing *Rhodococcus* from asymptomatic tissues. Published observations do not support their conclusion that bacteria lacking virulence genes isolated from asymptomatic tissues of symptomatic plants are pathogens (Stamler et al., 2015a). This can only be concluded if Koch’s postulates are reproducibly fulfilled.

In point iv, our statement that absence of data makes it challenging to disprove a hypothesis was interpreted as admission that there was no misdiagnosis (Randall et al., 2018). This is incorrect. We were acknowledging that even if deep sampling yielded no pathogenic strains, one cannot definitively exclude the possibility that *Rhodococcus* could cause disease to pistachio. However, based on the strains tested and the inability to reproduce findings, there is inadequate evidence to support the conclusion that *Rhodococcus* causes pistachio bushy top syndrome.

## Evidence that nonpathogenic isolates are benign/mutualistic (Vereecke, 2018)

*Rhodococcus* has been cultured from roots (Bulgarelli et al., 2012; Lundberg et al., 2012). Our study had the proper controls. We did not quantify lateral roots because the data were binary and changes in root hair number and length were more robust.

## Evidence for growth promotion by and virulence of PBTS isolates (Vereecke, 2018)

Many discoveries are made on the basis of studying one genotype. The findings that *fas* genes are necessary for virulence of *Rhodococcus* was on the basis of studying only D188. The most parsimonious explanation is that *Rhodococcus* cells lacking virulence genes are not pathogenic.

## Statements about PBTS (Vereecke, 2018)

There are a number of sources for our statement that ‘A second incidence of pistachio bushy top syndrome occurred in 2016’: these include Deckers, 2017 (which refers to ‘the second outbreak (started in summer 2016)'), Gutierrez and Francis, 2017, and the organized by the California Pistachio Research Board.

## Identity of D188-5 strain (Vereecke, 2018)

A second strain, also from Dr. Vereecke, has a deletion but in a different region of the chromosome (unpublished data). This second strain and the D188-5 we sequenced were from the same lineage that was purposefully subjected to stresses (Desomer et al., 1988). These strains were also maintained for ~25 years prior to us receiving them. Thus, the most parsimonious explanation is the deletion in the chromosome occurred prior to when we acquired D188-5.

## *Fas*-cytokinins and leafy galls (Vereecke, 2018)

BA is a synthetic cytokinin and is commonly used by researchers. Its resistance to cleavage by oxidases makes it appropriate for testing whether exogenously applied cytokinins can phenocopy the *Rhodococcus*-dependent disease symptoms (Kieber and Schaller, 2014). We used BA to demonstrate that 0.1 μM of cytokinin is necessary to inhibit root elongation to the same degree as 2.5 × 10^3^ cfu of pathogenic *Rhodococcus*. Given the low amounts detected in culture-grown *Rhodococcus*, we questioned whether pathogenic *Rhodococcus* could produce sufficiently high quantities of cytokinins to incite disease.

## Interpretation of observed phenotypes (Vereecke, 2018)

The *att* mutant and strains of *Rhodococcus* carrying the plasmid from the *att* mutant of D188 consistently caused leafy galls (Savory et al., 2017).

## Misrepresentation of previous work (Vereecke, 2018)

Points i, iv, and v misrepresent Savory et al. (2017) by neglecting the word ‘suggested’, by neglecting findings that non-pathogenic isolates were cultured from many species of nursery plants, and by neglecting other statements that recognized only three loci of the virulence plasmid are demonstrated to be necessary.

In response to point ii, in a presentation to stakeholders, slide 66 states ‘*fasD*: not recommended’ for PCR-based screening for *Rhodococcus* (Randall, 2015a).

Point iii is addressed above.

## Summary

We feel that we have addressed the comments of Randall et al., 2018 and Vereecke, 2018.
